# Same dish with new ingredients? —Implicit conceptions of first-year pre-service teachers about the role of emotions in learning processes

**DOI:** 10.3389/fpsyg.2025.1577048

**Published:** 2025-05-23

**Authors:** Rodolfo Bächler, Byron Quiroz, Pablo Segovia-Lagos, Macarena Otárola, Fernando Cofré

**Affiliations:** ^1^Escuela de Psicología, Facultad de Medicina y Ciencias de la Salud, Universidad Mayor, Las Condes, Chile; ^2^Escuela Reina de Suecia, Maipú, Chile; ^3^Facultad de Ciencias Sociales, Universidad de Chile, Ñuñoa, Chile; ^4^Carrera de Sociología y Carrera de Trabajo Social, Universidad Academia de Humanismo Cristiano, Santiago, Chile

**Keywords:** implicit conceptions, pre-service teacher, emotions, learning processes, beliefs

## Abstract

In the context of the emotional turn experienced in the world and in educational systems, this study undertook the task of examining the conceptions that pre-service teachers of first year have re-garding the role of emotions in educational processes. For this purpose, a dilemma questionnaire was applied to a sample of 72 first-year pre-service teachers from Chilean universities. The in-strument considered three types of conceptions called, in an increasing order of complexity, “behavioral reductionism,” “influence of emotions on cognition” and “cognitive emotional in-tegration.” The results show a rejection of the simplest and the most complex conception, and a large degree of adherence to the conception that separates emotions from cognitive processes, called “influence of emotions on education.” There are analyzed the differences found according to the gender of the students and a reflection on the possibility that the adherence to dualistic con-ceptions could be a way of reacting to an education based on fear as the only driving force for learning.

## Introduction

In recent decades, there has been a change in our conceptions of social life, which some have described as an “emotionalisation” of public affairs (Lara and Domínguez, 2013). This process is, in general, a change in our understanding of the role of affect in social phenomena, a change that has implications, for example, in the organizational world (Briner, 1999; Feldman and Blanco, 2006), in sport (Ros Martínez et al., 2013) and in politics (Nussbaum, 2006; Han, 2022). These cases, and many others that could be cited, are examples of a global phenomenon known as the “affective turn” which consists, broadly speaking, of a general awareness among social science researchers of the need to value affect as an important part of the studies and explanations they develop (Clough and Halley, 2007).

Educational world is no stranger to this change of focus, and in recent times, there has been a notable shift in the analysis of educational processes, with an increasing focus on the affective dimension. This has led to the emergence of a substantial body of literature on the subject (see Pekrun and Linnenbrink-Garcia, 2014), reflecting a growing interest within the field of educational research in understanding the role o emotions. However, as Saric (2015) has also observed, the proliferation of these studies does not necessarily imply a consensus among researchers on the conceptualization of emotion. Moreover, not only exist different conceptions about emotions within educational research, but also, as Fried et al. (2015) point out, there is an enormous amount of research that is conducted without being explicit regarding to what should be understood by “emotion” in each study. This problem has multiple etiologies, one of which is the intricate history behind the concept of “emotion” (Calhoun and Solomon, 1992; Campeggiani, 2023). Subsequently, once this concept has been established in our culture, it is subjected to investigation from disparate perspectives, each with a distinct emphasis on defining what an emotion is (Johnson and Arduiz, 2021). As outlined by Barrett et al. (2016), the field of emotional understanding can be approached from five principal disciplinary perspectives: biological, developmental, social and personality, cognitive, and health-related. Additionally, interdisciplinary approaches may also be considered. These approaches emphasize disparate aspects of emotions. Rather than representing opposing viewpoints, they can be understood as complementary perspectives that inform the design of an adequate study on conceptions of emotions. Nevertheless, in order to gain a more comprehensive understanding of this subject, it is essential to consider not only the theoretical perspectives espoused by researchers and experts on the topic of emotion, but also the everyday understandings held by the general public on this matter or what is referred in cognitive science as “folk psychology” (Ford and Gross, 2019). Research in this area has focused on two main issues, namely, beliefs about the positive or negative nature of certain emotions and, on the other hand, conceptions about the possibility or impossibility of exercising control over affects (Harmon-Jones et al., 2011; Tamir et al., 2007). It is notable that studies conducted in this area far have not addressed beliefs about the relationship between emotions and cognitive processes. This is particularly surprising given the significance of this topic, for instance, in the context of education. It is likely that this gap is in part due to the emotional-cognitive dualism^1^ that prevails in our culture (Duncan and Barrett, 2007), which has a major impact on the dominant ideas within our education systems.

Considering all of the above, in this study we will adopt a constructionist perspective on emotions (Bachler, 2019). This approach assumes that emotions are states integrated into so-called cognitive processes, so there is no separation between the two processes. Furthermore, from this perspective, the central component of emotions is their phenomenology, that is, the internal or subjective experience that occurs when experiencing an emotion. According to Barrett and Russell (2014), this phenomenology resides in core affect, that is, the ability to feel good or bad at varying degrees of intensity. Based on the above, behavior should not be understood as a central property of emotions since it would be merely a possible consequence of the emotional experience or core affect. This perspective, developed in the section entitled “Emotional-cognitive Integration,” is based on different studies in neuroscience and affirms that every teaching or learning process is ultimately an emotional process.

### Implicit conceptions about the role of emotions in educational processes

Recently, in the context of the emotional turn described above, a series of studies have examined the conceptions that teachers have about the role of emotions in teaching and learning processes (Bächler and Pozo, 2016; Bächler et al., 2018, 2020; Bächler and Pozo, 2020). It is about studies that adopt the view of conceptions as implicit phenomena, that is, as beliefs and assumptions with respect to different educational topics, and which those who hold them are not able to put into words, due to their non-conscious nature (Pozo, 2009). In addition, these works are framed within the assumption of a deep integration between emotions and cognitive processes, following several studies that have noted this point in neuroscience (Duncan and Barrett, 2007; Gu et al., 2012; Pessoa, 2013). The results of this studies made it possible to identify different conceptions that were organized in a progression from a simpler to a more complex perspective. Below is a summary of the main characteristics of the three conceptions.

#### Behavioral reductionism

Those who hold this perspective are people who equate emotions with behaviors, so that when they are asked about the emotions that are most common in the classroom, they express responses that refer to behaviors such as crying, laughing or shouting, among others (Bächler and Pozo, 2016). In coherence with this point of view, this conception was called “behavioral reductionism” since those who implicitly adhere to this approach do not seem to value the mental status of emotions by assimilating them to their associated behaviors. On the other hand, considering that this conception ultimately constitutes a denial of the existence of emotions, given their mental ontology, this perspective was characterized by the slogan “the absence of emotions in teaching and learning.” On the other side, in coherence with the reductionism that characterizes this conception, from this approach there is no appreciation of the value of emotions^2^ and their role in learning (Bächler and Pozo, 2020). In this case, all that is taken into account is the degree in which a behavior is more or less disruptive or adaptive for the learning objectives linked to a given educational situation.

#### Influence of emotions on cognition

The second perspective is one that does recognize the mental status of the emotions, understanding that they are states that have a subjective and qualitative nature that allows to differentiate them from other types of psychological processes such as learning (Bächler et al., 2018). Coherently with the above, from this point of view emotions are seen as the context of educational processes, in other words, as states that “influence or condition teaching and learning, as well as they do other contextual aspects, such as the temperature or the number of students in the classroom, for example” (Bächler et al., 2020, p. 79). Furthermore, the type of influence that emotions have on learning processes is conditioned, in this approach, by the valence of affects (Bächler et al., 2020). This means that “if an emotion is experienced as pleasant, then it favors learning, and if, on the contrary, it is experienced as unpleasant, then it is an obstacle to teaching and learning” (Bächler et al., 2018, p. 766).

#### Emotional-cognitive integration

The two conceptions described in the previous sections correspond to perspectives on the role of emotions in educational processes that are based on a excision between affects and processes traditionally considered as “cognitive.” This is a way of seeing things that is deeply rooted in our culture and that contrasts with the third perspective identified, called “emotional-cognitive integration.” From this latter conception, emotions are considered as the center of teaching and learning processes, without drawing ontologically substantive distinctions between both types of processes (Bächler et al., 2018). This is a point of view that is coherent with different findings in neuroscience that show a narrow interweaving between affectivity and cognition in the brain (see, for example, Damasio, 2010; Duncan and Barrett, 2007). The statement of the Spanish neuroscientist Francisco Mora reflects this idea very well when he explains: today we are beginning to know that the binomial emotion-cognition (mental processes) is an indissoluble binomial. And this is due to the design of the brain and how it works. The abstracts, the concepts that the brain creates, are not aseptic of emotion, but impregnated with it. This should alert us to the importance of emotion for both the learner and the teacher (Mora, 2013; p. 38). Even when, in everyday experience, human beings have the feeling that learning is a strictly cognitive phenomenon, in the representational sense of the term, it would ultimately have other characteristics. From this perspective, learning can be understood as a process that starts under the form of affective states which are subsequently re-shaped until they acquire a symbolic representational character and could eventually be communicated (Bachler, 2019). Given the processual nature of learning, which consists of a transition from the emotional to the symbolic-representational, the valence of affects does not become in a property that is relevant to determine the characteristics of this process. From this perspective, it is possible to learn through experiences as dissimilar as fear, joy or shame, since the process, rather than being influenced by emotions, is itself emotional.

The conceptions described above were evaluated with samples of primary school teachers (Bächler and Pozo, 2020) and with a group of university teacher educators (Bächler et al., 2023). In both cases a strong attachment to the “influence of emotions on cognition” conception was found. Although in the case of the teacher educators this tendency was shared with the “cognitive emotional integration” conception. However, despite all this advancement, we still do not have information on the conceptions that pre-service teachers have on this subject at the time of starting their studies. This is a relevant weakness since the existing literature suggests that initial teacher education (ITE) is the privileged space for modifying viewpoints about the role of affects in educational processes (Bächler et al., 2020; Kelchtermans and Deketelaere, 2016). This process requires, in first place, to know what conceptions students have at the time they start their studies. This is because any intentional change must be made based on the existing conceptions, as pre-service teachers are not an empty box to be filled during ITE. On the contrary, they come to training with a set of ideas about education that are the result of observational learning processes during their passage through the school system (Lortie, 1975). Rescuing those ideas is therefore a necessary condition for any change process since learning always occurs on the basis of what already exists (Contreras, 2016).

On the other hand, it is interesting to know whether students' conceptions differ depending on the type of university in which they are enrolled. Of course, if there are differences, these cannot be attributed to the type of education the university provides, since these are first-year students. However, in Chile, in recent decades, there has been an explosive decline in new private universities, and there is a strong debate regarding their quality and the understanding they maintain of human development and vocational training. This fact contrasts with the view of state universities as civic institutions closely linked to the country's development (Monckeberg, 2005, 2007, 2013).

Finally, it is relevant to wonder if the conceptions that pre-service teachers have when they start their studies are in any way related to their gender. This is a question that can be supported by an existing background in literature that shows differences between men and women on issues related to emotions. For example, some studies claim that there are differences in the learning outcomes of girls and boys in the school system, which are attributed to the fact that female students internalize a certain degree of anxiety from their female teachers, a mechanism based on the belief of gender stereotypes (Antecol et al., 2012). Other studies show links between emotional intelligence and gender, a relationship that generally favors female teachers over their male peers (Chan, 2004; Gürol et al., 2010; Rastegar and Memarpour, 2009). Finally, and only with the aim of showing some background information on this issue, there are studies that show differences in the expression of emotions between men and women, which result in a greater expressiveness of positive emotions, along with a greater internalization of sadness by women (Chaplin, 2015).

In the context of all the above, the present study aims to solve the following research questions:

(1) Which are the implicit conceptions about the role of emotions in educational processes that are most preferred by pre-service teachers of first year at Chilean universities?

(2) Which are the implicit conceptions about the role of emotions in educational processes that are most rejected by pre-service teachers of first year at Chilean universities?

(3) How is the relationship between the preferred or rejected conceptions and the type of university in which they study and the gender of the students?

### Methodology

The aim of this study was to describe the conceptions of first-year pre-service teachers about the role of emotions in teaching-learning processes. It was used a quantitative approach under an exploratory and descriptive design (Johnson et al., 2007).

### Sample

For this study, it was constituted a non-probabilistic sample by convenience made of 72 first-year student teachers from different Chilean universities, both state and private, and from different regions of the country. Table 1 shows the characteristics of the sample and its composition.

**Table 1:** Composition of the sample.

**Gender**		**Type of University**		
**Female**	**Male**	**Other**	**Private**	**State**
44	26	2	49	23
61.1%	36.1%	2.8%	68.1	31.9

### Instrument

For the examination of the conceptions, it was applied to all the participants a dilemma questionnaire which had already been used successfully in several previous research studies (Bächler et al., 2020, 2021, 2023). The instrument is composed of eight items, each of which presents an educational dilemmatic situation.

For each dilemma, there are offered three response options and participants were asked to mark the option they liked the most and the option they liked the least. This classification allows to tabulate the responses by assigning a score of +1 for the preferred option and −1 for the option they liked the least; the option left in blank is scored with a value of zero.

Each option of answer is associated with one of the following conceptions about the relationship between emotions and teaching-learning processes: Behavioral Reductionism (BR), Influence of Emotions on Cognition (IEC) and Emotional-Cognitive Integration (ECI).

The following is a presentation of one of the dilemmas of the questionnaire and its response options in order to illustrate the characteristics of the instrument.

*The students are in the science class working in the laboratory. During the session, the teacher notices that some students are restless due to their enthusiasm*^3^ *for experimenting with chemical transformations of matter. Facing this situation, the teacher decides:*

(a) Regulating the behavior of learners to avoid disrupting the teaching-learning process (BR conception).

(b) Congratulate students for their enthusiasm and use their emotions to strengthen teacher-student relationships (IEC conception).

(c) Use enthusiasm to deal with some aspects of science experimentation, even if it takes some extra time to the development of other contents (ECI conception).

### Plan of data analysis

The processing and data analysis was made using the statistical software SPSS version 24. Firstly, descriptive analyses were carried out in order to characterize the sample and to analyse the means with respect to the degrees of acceptance and rejection of the conceptions expressed by the student teachers about the role of emotions in the teaching-learning process; for this purpose, non-parametric tests were used, since an atypical behavior was observed in the sample (*p* > 0.05).

Secondly, due to the exploratory and descriptive nature of the study, in addition to the small sample size, it was carried out a K-means cluster analysis the existence of groups of participants who had in common differentiated response patterns that could be interpreted as conception profiles. For this purpose, it was considered as a variable the total of answers given by each participant in the study to each of the conceptions. Once the groups had been identified, an analysis of corrected typified residuals was done with the aim of finding out if they were linked or not to any particular gender.

### Ethical considerations

This research was conducted in accordance with the rules of the declaration of Helsinki of 1975 and revised in 2013, it was also approved by the scientific ethics committee of Universidad Mayor, the sponsor of this study, under resolution of the act n° 0242 of 26-01-22. All the students participated voluntarily by signing an informed consent form, which guarantees the anonymous character of the data processing and the exclusive and confidential use of these data for the purposes of this research.

## Findings

### First phase: analysis using the *z*-test of Kolmogorov-Smirnov

Table 2 shows the findings obtained in this phase.

**Table 2:** Findings obtained from the application of the Kolmogorov-Smirnov test.

	**BR mean**	**IEC mean**	**ECI mean**
Asymptotic Sig. (bilateral)	0.022	0.055	0.004

As it can be seen, of the three variables considered in the analysis (degree of acceptance or rejection of the BR conception, degree of acceptance or rejection of the IEC conception and degree of acceptance or rejection of the ECI conception) only one (IEC) has the form of a normal distribution. For this reason, it was decided to conduct all the analyses with non-parametric statistics.

### Second phase: determination of the degrees of acceptance or rejection expressed for each conception

**Figure 1:**
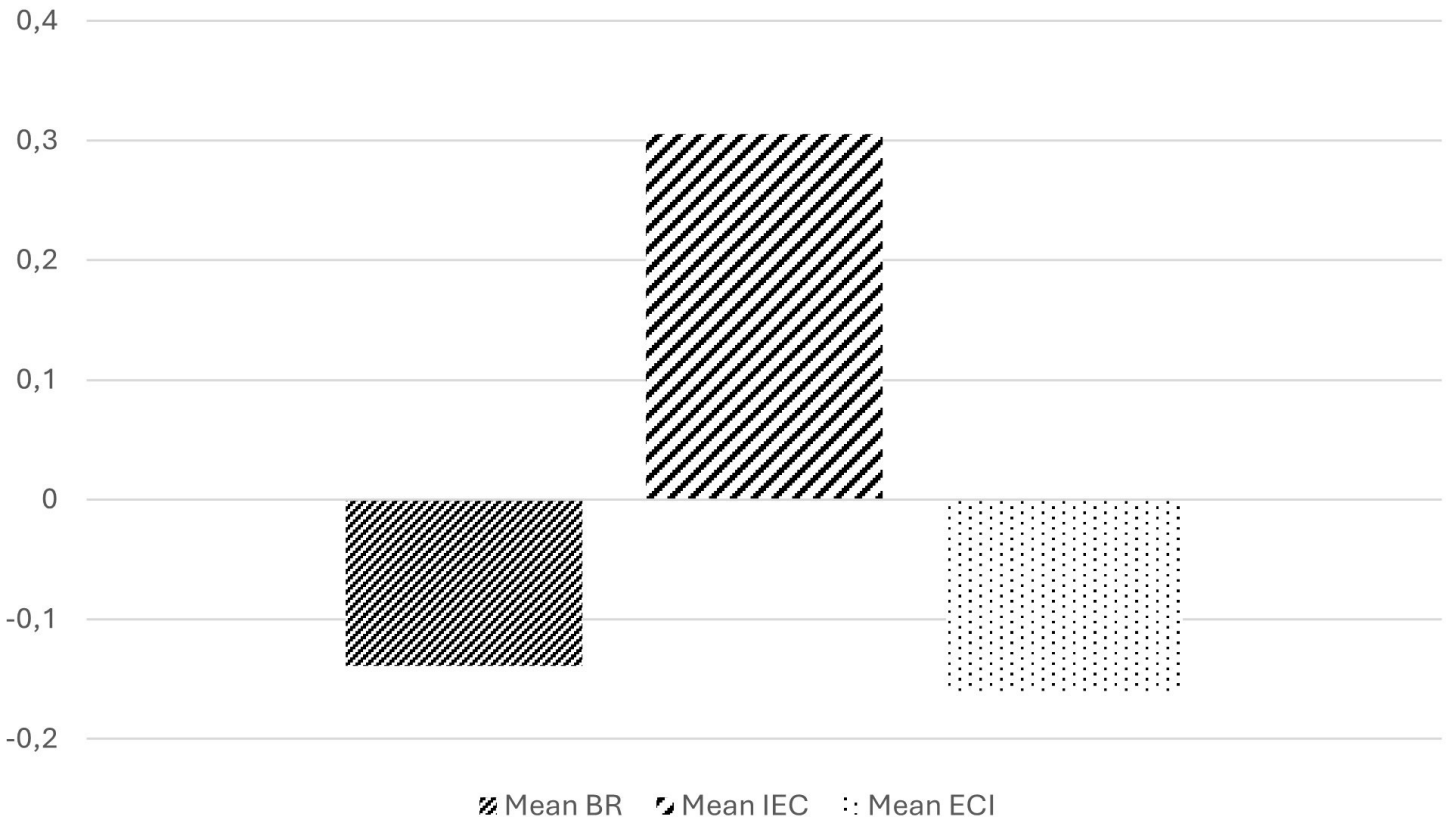
Mean of acceptance and rejection of each conception in the complete questionnaire.

As can be seen in Figure 1, the conception “Influence of emotions on cognition” is the one preferred by the participants in the study. It is, in addition, the only conception for which a positive mean score is expressed, denoting an acceptance of this perspective (x̄ = 0.3056). With respect to the other two conceptions, on the contrary, the mean scores obtained are negative, showing rejection of both perspectives (BR x̄ = −0.1389; ECI x̄ = −0.1667). This means, according to what has been analyzed in the previous sections, that most of the participants in the study conceive the existence of a separation or dichotomy between emotions and cognitive processes, assuming that affects play a role as a context for the teaching and learning processes. Furthermore, from this point of view, which is pre-eminent among the participants in the study, it is considered that emotions of positive valence are intrinsically facilitative of teaching and learning processes while the emotions of negative valence are conceived as an obstacle to these processes.

On one hand, the conceptions “Behavioral reductionism” and “Emotional-cognitive integration” are both rejected without significant differences in the degree of intensity of this rejection (Wilcoxon: *Z* = −0.343, *p* = 0.732). The above allows to affirm, in first place, that for the majority of the participants in the study it is inconceivable to deny the mental status of emotions and their role in teaching and learning processes just as it is implicitly held the BR conception. On the other hand, the results regarding the ECI conception allow to understand that the rejection expressed with regard to the possibility of reducing emotions to their associated behaviors does not mean in any case a recognition of the possibility of integration between emotions and cognitive processes.

The results presented above provide a general overview of the degrees of preference or rejection expressed for each of the conceptions by the total group of participants. However, as anticipated, for a deeper understanding of the particularities of each point of view, a cluster analysis was carried out, and its findings are described below.

### Third phase: identification of groups of participants with differentiated responses and interpretation of these responses in terms of profiles of conceptions

After performing analyses considering different numbers of groups, it was decided to define three groups to carry out the cluster analysis. The decision was made taking into account two criteria. The first was to arrive at a number of groups that presented clear differences in terms of their degrees of acceptance or rejection of the different conceptions, which could be explained theoretically. The second was to consider groups of relatively similar size.

Table 3 shows the resulting clusters of the analysis, highlighting with a more or less dark gray background the values that denote a preference for some of the conceptions.

**Table 3:** Resulting clusters from the classification with all the responses to the questionnaire.

	**1 Behavioral reductionism**	**2 Influence of emotions on cognition**	**3 Transition from the influence of emotions on cognition to the emotional-cognitive integration**
Mean C1 behavioral reductionism	0.19	−0.19	−0.37
Mean C2 influence of emotions on cognition	0.01	0.57	0.22
Mean C3 emotional-cognitive integration	−0.20	−0.38	0.15

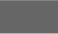
 color means a strong preference for a specific conception. 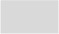 color indicates a moderate preference for a specific conception.

#### Group 1 composed of 20 participants. Profile “behavioral reductionism: the absence of emotions in teaching and learning processes”

This is the smallest of all the groups identified. It is characterized by expressing a preference for the BR conception accompanied by a neutral attitude toward the IEC conception and a rejection of ECI. Because of these results, this profile was called “Behavioral reductionism: the absence of emotions in teaching and learning processes.” This is a perspective that implicitly denies the involvement of emotions in teaching and learning by assimilating the affective world to its associated behaviors. On the other hand, although this profile is not completely closed to the possibility of considering emotions as a context for cognitive processes, it does reject absolutely considering any kind of integration between affects and learning processes.

#### Group 2 composed of 29 participants. Profile “influence of emotions on cognition: emotions as a context for cognitive processes”

This is a group of participants whose common characteristic is their preference for the IEC conception and an open rejection of the other two conceptions considered in the study (BR and ECI). This implies, on one side, the assumption of the fact that emotions are mental states that cannot be reduced to their associated behaviors, but on the other side, the rejection of the possibility of integrating affects and cognitive processes. What is argued here is that emotions, although they are states different from cognitive processes, have nonetheless an influence on the latter. Specifically, from this perspective it is argued that emotions influence teaching and learning processes by virtue of their valence. This means that if the affects that participate in an educational situation are of positive valence, then they become facilitators of the teaching and learning processes. In contrast, if these emotions are of negative valence, then they constitute an obstacle for teaching and learning.

#### Group 3 composed of 22 participants. Profile 3 “transition from influence of emotions on cognition to emotional-cognitive integration”

The participants grouped under this cluster express rejection of the possibility of reducing emotions to their associated behaviors and, in parallel, express adherence to the IEC conception (with more intensity) and ECI (with less intensity). Due to this double orientation, the responses of this group have been interpreted as a profile of “Transition from the Influence of Emotions on Cognition to Emotional-cognitive Integration.” This would be a group of participants that, in most of the cases presented in the questionnaire, adopts an IEC perspective but that, nevertheless, in some cases, tends to “progress” toward an ECI perspective. This “progress” means an overcoming of emotional-cognitive dualism and the assumption that any emotion, regardless of its valence, can be configured as the beginning of the teaching and learning processes.

Subsequently, once the profiles had been identified, it was carried out a chi-square analysis with the aim of finding out if any of the socio-demographic conditions considered in the study (type of university where they studied and gender) were differentially represented in each of the profiles. Regarding the type of university, no differences were found, whereas in the case of gender, there were (χ^2^ = 9.983, *df* = 2, *p* < −0.087). Subsequently, in order to identify what form these differences would take, it was carried out an analysis using corrected standardized residuals, and the results can be seen in Table 4, highlighting in gray the values that are above the expected.

**Table 4:** Corrected typed residuals resulting from relating the profile belonging to the gender of the participants.

			**Profile 1**	**Profile 2**	**Profile 3**	**Total**
Gender	Female	Recount	7	23	13	43
		Corrected residual	−3.0	2.5	0.3	
	Male	Recount	13	6	7	26
		Corrected residual	3.0	−2.5	−0.3	
Total	Recount		20	29	20	69

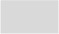
 color means a proportion of cases below or above what is expected.

As it can be observed, there is a higher proportion of females than expected in Profile 2 “Influence of emotions on cognition: emotions as a context for cognitive processes.” At the same time, there is a higher presence of males in Profile 1 “Behavioral Reductionism: the absence of emotions in teaching and learning processes.”

## Discussion and conclusions

In first place, it is necessary to highlight that this study has found that the conception called “behavioral reductionism” is rejected by the participants in the research. In general, it seems to pre-service teachers of first year that ignoring the mental status of emotions and focusing their attention exclusively on the behavior of students is not a good idea. This is, at first sight, a positive piece of news, since it implies a step forward in relation to the behaviorist positions that have historically prevailed in our school cultures. It is, additionally, an advance that is consistent with the fact that the conception that generates the highest levels of adherence among the participants in this study is the “influence of emotions on cognition” (IEC), a point of view that recognizes emotions as mental states that are different from behaviors. However, as analyzed in the foundation of this study, IEC corresponds to a dualistic perspective, which establishes a separation between emotions and teaching and learning processes. Moreover, from this point of view, it is assumed that some emotions are intrinsically favorable to educational processes, while others are in themselves an obstacle to learning. Both characteristics of this perspective are aspects that are not in line with the most recent scientific findings on the functioning of the mind. For the same reason, it can be concluded that the advance toward the recognition of emotions as mental states different from behaviors does not translate itself into a true overcoming of the emotional-cognitive dualism that prevails in our culture, which was mentioned in previous sections of this article. In fact, the so-called “emotional-cognitive integration” conception receives similar levels of rejection as those expressed for behavioral reductionism. Which could be the reasons behind these results? It is likely that the paradigm of emotional education, constituted by the emotional intelligence discourse, hegemonic in education (Menéndez Álvarez-Hevia, 2018), as well as the growing presence of the positive psychology perspective (Cabanas and González-Lamas, 2021), are two factors that contribute to strengthening this perspective among pre-service teachers. In this sense, it seems that the currently dominant emotional perspective in our educational systems (Bächler and Pozo, 2020; Bächler and Salas, 2021; Bächler et al., 2023) probably is one that emerges reactively to decades of preeminence of a “spare the rod and spoil the child” type of education, characterized by the presence of different types of suffering in school. In this regard, the study carried out by Schohaus (1930/2012) almost a century ago showed different dimensions of the suffering that schools caused in their educational communities. This study has a line of continuity in the research of Polizzi and Frick (2024), which identifies new facets of school suffering today. It is notable that in the face of the different forms of expression that suffering takes in schools, these last authors present as examples of good responses to this problem different ways of compensating for the effects of punitive education through strategies and classroom climates based on the idea of happiness (the happiness curriculum in India, socio-emotional learning in the United States, and the trauma-informed curriculum (Ginwright, 2018).

In this new scenario, it is assumed that emotions of positive valence are the only possible type of affectivity for the achievement of educational goals (Cabanas and Illouz, 2019). If all of the above is correct, since these are speculations that need to be explored empirically, the findings of the present study rather than “telling us” about a shift of paradigm, in the sense of questioning the epistemological assumptions on which the new perspective is built, probably reflect the replacement of a type of emotionality of negative valence by another of positive valence. The underlying assumptions remain intact, however, since it is still assumed that there is a dichotomy between emotional and cognitive processes. Therefore, there is still a narrow view that limits the emotional wealth of educational processes.

With regard to the sociodemographic variables considered in the study, as has already been pointed out, there were no differences in the degrees of adherence or rejection to the conceptions according to the type of university to which the students belonged. Given that these were first-year students, this result is expected, since the necessary time has not yet elapsed for the training to have the possibility of modifying conceptions. Nevertheless, the results in this area suggest, on the other hand, that there is no profile of underlying conceptions, before starting the studies, that is associated with the choice of a university, public or private, in which to train professionally.

This result is probably also related to the limited development of the topic of initial emotional teacher training, a situation that makes it impossible to differentiate between institutions regarding this dimension. With regard to the other variable considered in the analysis, the gender of the students, it is remarkable that the preeminence of the conceptions is not equal between men and women. The results of cluster analyses show that men are more present in the profile called “behavioral reductionism,” while women are more represented in the profile called “influence of emotions on cognition.” It can be said, then, that the change of perspective, in the sense of recognizing the role of emotions in educational processes, as proposed by approaches such as emotional intelligence, social-emotional learning, positive psychology, and others, has had a greater impact on women. In fact, the concept of “The Influence of Emotions on Cognition” is a way of looking at the role of emotions in educational processes that incorporates some of the main postulates of the aforementioned approaches. Men who enter the teaching profession, on the contrary, remain more attached to an education with behavioral characteristics. It will be a subject for further studies to corroborate this finding and investigate its possible causes. For the moment, it can only be hypothesized that this difference has a sociocultural origin, which would be consistent with the constructionist perspective of emotions.

Although the cluster analysis identifies, on the other hand, the existence of a group of participants who are in a transition from a dualistic point of view toward a perspective that integrates emotions and cognition within educational processes, it is not known at the moment the specific characteristics of this group as well as the factors that may facilitate this step. However, it seems obvious that ITE should play an important role in terms of catalyzing this transition toward more integrative positions. Nevertheless, the literature reviewed is not very auspicious for this shift. What is known so far, with the only study conducted in this area, is that those teacher education institutions that follow the precepts of emotional education do not have an impact in terms of generating deep emotional change among their students (Bächler et al., 2020). Therefore, along with recommending the evaluation of ITE processes, this study encourages further research that takes into account different levels of evaluation in the implicit/explicit continuum of conceptions in order to determine at what level changes occur, in case there are any.

Finally, this article ends by mentioning an identified limitation of the study. This is the fact that we have had a small sample consisting of only 72 students from different Chilean universities. In this context, its findings should be considered as a first approximation to the task of identifying the conceptions that teaching students have on this subject. This study will have to be endorsed, however, with new research that expands and diversifies the sample of participants. In the same line, it would be important to know if these findings are consistent with the educational reality of other countries, considering the influence that different cultures could exert on this field.

## Data Availability

The raw data supporting the conclusions of this article will be made available by the authors, without undue reservation.

## Footnotes

^1^Cognitive emotional dualism is the belief that emotions and cognitive processes exist as clear and distinct natural entities. It is the affirmation of the existence of such states in their ontological sense.

^2^The valence of emotions corresponds to the fact that, subjectively, these states are experienced as something pleasant or unpleasant.

^3^This item refers to positive valence emotions. Other items, on the other hand, were designed with the objective of evaluating the conceptions that the participants in the study have about the participation of negative valence emotions in educational processes.
